# Characterization of differences in volatile compounds and metabolites of six varieties of potato with different processing properties

**DOI:** 10.1016/j.fochx.2024.102116

**Published:** 2024-12-22

**Authors:** Wenyuan Zhang, Liang Li, Yaqi Zhao, Haixia Yang, Xuejie Zhang, Zhanquan Zhang, Xue Wang, Zhenzhen Xu, Wanxing Wang, Jianjun Deng

**Affiliations:** a State Key Laboratory of Vegetable Biobreeding, Institute of Vegetables and Flowers, Chinese Academy of Agricultural Sciences, Beijing 100081, China; b College of Food Science and Nutritional Engineering, China Agricultural University, Beijing 100083, China; c Institute of Quality Standard & Testing Technology for Agro-Products, Key Laboratory of Agro-food Safety and Quality, Ministry of Agriculture and Rural Affairs, Chinese Academy of Agricultural Sciences, Beijing 100081, China

**Keywords:** Potato, Fresh eating, Food processing, Volatile compound, Metabolites

## Abstract

Potato is the fourth-most important food crop around the world, and most of the potatoes are used for foodstuffs and starch products. The aim of this paper is to identify the volatile compounds and metabolites in potatoes with different processing properties. The results showed large differences of volatile and metabolite compounds such as 2,4-Heptadienal and rhoifolin in potatoes and indicated the potential regulations between volatile compounds and metabolites. Moreover, the differences in volatile and metabolite compounds were compared between fresh eating and processing type potatoes. Compared to process type potatoes, fresh eating potatoes contained a higher proportion of aldehyde and alcohol compounds, but being lower in hydrocarbon, furan, and ketone compounds. Moreover, the different expressed metabolites were involved in the metabolism of amino acids, flavone and flavanol biosynthesis, and tryptophan metabolism. The Random forest showed that the fresh eating and processing type potatoes could be distinguished by the content of amino acids and phenols.

## Highlights

• The volatile and metabolite compounds are significantly different in potatoes.
• The metabolites play important roles in the volatile compounds in potato.
• Amino acid is the important variable to identify the processing property of potatoes.
• This study provides a novel insight into breeding strategies for potato varieties for different processing applications.

## 1 Introduction

Potatoes are the tubers of the plant *Solanum tuberosum* L., which originated from the Andes Mountains in South America. For now, the potato has been considered as the fourth-most important food crop following maize, wheat, and rice in the world with a production amount of 377 million tons (Tunio et al., 2020). China has been the largest potato producer among the world since 1993, which contributes about 22 % to the world production followed by India, Ukraine, and USA. There are four major potato producing regions in China contributing to 60 % of China's total fresh potato production and Sichuan, Gansu, Guizhou, Yunnan, and Inner Mongolia are the largest potato-producing provinces among these regions. Currently, in China, most of potatoes (about 45 %) are used for foodstuffs, 21 % for starch and other processed products, 14 % for export and feed (Wang et al., 2023).

The flavor and taste of potatoes are becoming increasingly importance to consumers. However, there are still limited studies reporting the complex flavor traits and their relationships with their components (Morris et al., 2010). Generally, the dominant volatile compounds in potatoes include 2-octenal, hexanal, heptanal, pentanol, 2-pentylfuran as well as 2-methyl- and 3-methylbutanol (Dresow & Böhm, 2009). Significant differences in volatile compounds have been observed among potatoes from different areas (Y. Wu et al., 2023). Potatoes contain about 80 % water and 20 % dry matter, with the most abundant in carbohydrates followed by protein. Of these, potato starch constitutes about 70 % to 85 % of the potato dry matter and mainly consists two types of starch which are lightly branched amylose and highly branched amylopectin (Reyniers et al., 2020). Variations in the structures of potato starch result in differences in its functional properties such as gelation, texture, and viscosity compared to cereal and other starches (Reyniers et al., 2020). Due to the unique properties of potato starch, it leads to the larger application amounts of potato starch in food industry as a thickener, colloidal stabilizer, gelling agent, bulking agent, water retention agent and adhesive (Singh et al., 2002), and these potato cultivars have been considered as processing type potato. In addition to the starch processing, the processing type potatoes are normally freeze-dried and used for French fries, potato chips, and other pre-fried potato products (Keijbets, 2008). Moreover, potato is a highly nutritious crop with various nutrients such as vitamins, niacin, pantothenic acid, minerals, polyphenols, and flavonoids (Bnv & Gvs, 2023), and some potato cultivars belong to fresh eating type potatoes. Previous study has reported that potato contributes to the highest total phenolic daily intake among vegetables (Miranda et al., 2013). Potatoes contain various types of polyphenols including phenolic acids, flavonoids, and anthocyanins. Among these, the dominant phenolic acids in potatoes include chlorogenic acid, caffeic acid, ferulic acid, and gallic acid and the main flavonoids are kaempferol, quercetin, and myricetin (Rasheed et al., 2022). The health benefits of potatoes have been reported to include antioxidant, anticancer, antihyperlipidemic, antivascular, and antihypertensive effects to human (Sahani et al., 2023).

Recently, LC-MS/MS-based metabolomics approach has become a fundamental high-throughput tool, characterized by its higher sensitivity and selectivity including targeted and untargeted strategies (Bozza et al., 2024). Among these, untargeted metabolomics can comprehensively detect metabolites with molecular weights below 1000 Da from biological systems, and identify the relevant role of metabolites in various processes (Wolfender et al., 2015). Therefore, LC-MS/MS-based untargeted metabolomics has been widely applied as a powerful method to investigate metabolite variations in samples under different conditions (Li et al., 2023). Although variations in starch composition between fresh eating and processing type potatoes are well-studied, the detailed variations in volatile compounds and metabolite compositions still remain unknown. Accordingly, the aim of this study is to identify the volatile compounds and metabolites in different potato varieties with distinct properties, and explore the potential metabolite variations in fresh eating and processing type potatoes. To achieve this, six varieties of potatoes were collected from the same area, volatilomic and metabolomics analysis was performed to identify the volatile and metabolite compounds and further discussed their correlations with each other. Together, these results will provide a better understanding of the relationships between basic nutrients, volatile compounds, and metabolites with different processing properties. This work also helps to establish a foundational database for future potato breeding efforts.

## 2 Material and methods

### 2.1 Material

Six varieties of potatoes including Jingzhangshu 1(J1), Jizhangshu 8 (J8), Jizhangshu 12 (J12), Luxinda (V7), Qingshu 10 (Q10), and Xisen 6 (XS) were kindly provided by the cooperator of Fuzhong Potato Industry, Shanxi Province in China. All the commercially chemicals were analytical reagent and some chemicals were chromatographic grade for GC–MS and LC-MS/MS analysis. Sodium hydroxide, methanol, acetone, sodium chloride (NaCl), potassium hydroxide, hydrochloric acid, vitamin C, oxalic acid, ammonium molybdate, Ethylenediaminetetraacetic acid (EDTA) was supplied by Xilong Scientific Co., ltd. (Shanghai, China). Folin- Ciocalteu reagent, sodium carbonate, amylopectin, amylose, and gallic acid were purchased from Shanghai Yuanye Biotechnology Co., ltd. Sulfuric acid, acetic acid, ethanol was purchased from Beijing Tong Guang Fine Chemical Co., ltd. metaphosphoric acid, iodine was purchased from Macklin (Shanghai, China). The internal standard 2-Methyl-3-heptanone was purchased from Solarbio (Beijing, China).

### 2.2 Analysis of basic nutrients

The identifications of basic nutrient were followed the previous study including total polyphenol (Hung & Yen, 2002), vitamin C (Xu et al., 2021), and starch content (Zhou et al., 2014) with some modifications. Each basic nutrient for potato sample was measured in triplicate. The Folin-Ciocalteu assay is a reference method for the quantification of total (poly)phenols in food via redox reaction (Pérez et al., 2023), and gallic acid were used as standard to identify total polyphenol content in potatoes. One gram of dried sample was mixed with 20 mL of 80 % acetone solution and gently shook for 10 min, and passed through a 0.22 μm filter twice. Afterwards, Folin phenol reagent was added to 0.5 mL of standard solution or sample phenolic extraction solution. Then, 3.0 mL 10 g/100 mL sodium carbonate solution was added and adjusted the volume to 5.0 mL, and reacted in the dark for 30 min. Subsequently, the total volume of mixtures was fixed to 5.0 mL and reacted in the dark for 30 min. After reaction, based on the standard curve, the total polyphenol content was calculated using the absorbance values of the standard and sample solutions at 760 nm.

The identification of vitamin C was started with the mixture of 1 g dried potato with 5 mL 0.05 M oxalic acid and 0.2 mM EDTA solution. The mixture was centrifuged at 13000*g* at 4 °C for 20 min and the supernatant was collected. Afterwards, 2 mL supernatant was mixed with oxalic acid -EDTA solution, 0.5 mL metaphosphoric acid - acetic acid solution, 1 mL 5 % sulfuric acid, and 2 mL 5 % ammonium molybdate solution. The mixture was incubated at 80 °C for 10 min and measured the absorbance at 760 nm.

As for measurement of starch, 100 mg dried potato sample was mixed with 1 mL ethanol and 9 mL 1 M potassium hydroxide solution. The mixture was heating at 95 °C for 10 min and after cooling, the volume of mixture was adjusted to 100 mL. Subsequently, 2 mL abovementioned sample solution was mixed with 6 mL potassium hydroxide solution, 2 mL acetic acid, and 1 mL iodine solution, and left to stand at room temperature for 30 min. The absorbances were measured at 427 nm, 535 nm, 630 nm, and 757 nm, respectively. The concentrations of amylose and amylopectin solution ranging from 0.0 to 36.0 μg/mL were used for standard curves. The concentrations of amylose and amylopectin in potato samples were calculated as follows:

$$X_{1}=\frac{C_{1}\times 50\times D}{m\times 1000}\times 100$$

$$X_{2}=\frac{C_{2}\times 50\times D}{m\times 1000}\times 100$$

where X_1_ is the concentration of amylopectin in potato sample (g/100 g); X_2_ is the concentration of amylose in potato sample (g/100 g); C_1_ is the concentration of amylopectin in sample solution (μg/mL); C_1_ is the concentration of amylose in sample solution (μg/mL); D is the dilution factor; m is the sample weight (mg).

### 2.3 Sample preparation and GC–MS analysis

The volatile components of all potato samples were measured by solid-phase microextraction-gas chromatography and mass spectrometry (SPME-GC/MS) (7890-5985C, Agilent, USA) followed the protocol reported by Blanda et al. (2010) with some modifications. Briefly, 3 g potato sample was mixed with 0.3 g Nacl into an empty 20 mL headspace vial. An internal standard method was used for semiquantitative analysis with 0.5 μL 0.816 μg/μL 2-Methyl-3-heptanone. Afterwards, the headspace vial was with a silicon cap and heated at 50 °C for 30 min. Then, the divinylbenzene/carboxen/polydimethylsiloxane (DVB/CAR/PDMS, 50/30 μm, coating 2 cm) manual holder (Supelco Ltd., Bellefonte, PA, USA) was used to extract at 50 °C for 40 min. The column DB-WAX (30 m × 250 μm × 0.25 μm) was used with the flow rate at 5 °C /min, and temperature gradient program was as follows: 40 °C for 3 min; 40 °C - 200 °C for 5 min; 200 °C – 230 °C for 3 min; 230 °C for 3 min. The mass spectrum conditions were performed as follows: electron ionization source; electron energy 70 eV; ion source temperature 230 °C; transfer line temperature 280 °C; four-stage rod temperature 150 °C; mass scanning range *m*/*z* 55–500. The qualitative analysis for volatile compounds was performed with two methods (NIST library and retention index, RI), and the RI value (>70 %) for volatile compounds was selected. Each potato variety was repeated in triplicate. The quantification analysis for identified volatile compounds was calculated as follows:

$$C=\frac{S}{S_{i}}\times \frac{C_{i}}{m}$$

where C is the concentration of identified compounds (μg/g); C_i_ is the concentration for the internal standard; S is the peak area for identified compounds; S_i_ is the peak area for the internal standard; m is the sample weight (g).

### 2.4 Sample preparation for metabolomics

The sample preparations of potato for metabolomics analysis was followed with previous study with some modifications (Zhao et al., 2024). The potato samples were frozen dried, the ground by a high-speed blender and filtered by a 60-mesh filter. Subsequently, 1 g dried potato was added to 10 mL pre-frozen methanol solution (70 % *v*/v), and the ultrasound was performed for 30 min. The mixture was stored at −20 °C for 10 min, and centrifuged at 10000*g* for 20 min. The supernatant was collected and adjusted the volume back to 10 mL, and passed through 0.22 μm filter for analysis. The quality control (QC) samples were equally mixed with all samples to make sure the accuracy of produced metabolomics data (Kirwan et al., 2022). The metabolomics analysis for each potato variety was repeated in six times.

### 2.5 UHPLC-QTOF-MS for metabolomics

The UPLC separation (ExuinLC AB SCIEX, Massachusetts, United States) with Analyst TF 1.8.1 Software was utilized an ACQUITY UHPLC HSS T3 chromatographic column (1.8 μm × 2.1 μm × 100 mm). A sample injection volume of 10 μL was used for sample, regent blank, and QC sample, with the column temperature set to 40 °C. The prepared QC sample was injected after every five samples to ensure the accuracy of the instrument and batch consistency. The mobile phases consisted of water containing 0.1 % formic acid (A) and acetonitrile with 0.1 % formic acid (B), delivered at a flow rate of 0.30 mL/min. The gradient elution for LC separation was programmed as follows: 95–70 % A from 0.00 to 11.50 min, 70–0 % A between 11.50 and 11.51 min, 0 % A from 11.51 to 15.00 min, then 0–95 % A at 15.00–15.01 min, and 95 % A from 15.01 to 18.00 min. Mass spectrometry analysis was conducted using QTOF-MS (6600 SCIEX, Massachusetts, United States) in both positive and negative ionization modes. A full MS scan was performed across an *m*/*z* range of 75–1125, with a resolution of 70,000. For the mass spectrometry information-dependent acquisition (IDA), both primary precursor ions and high-sensitivity secondary product ions were recorded. The precursor ion scan range was 100–1000 Da, while product ions were scanned from 50 to 1000 Da. The QTOF-MS parameters included a temperature of 500 °C, ion source gas temperatures of 50 °C for both gases 1 and 2, and a curtain gas temperature of 25 °C. The ion spray voltage was set to 5.5 kV, the declustering potential to 60 V, and the collision energy to 10 eV.

### 2.6 Data analysis

The metabolomics raw data analysis from UPLC-MS/MS was analyzed by MS-DIAL software (version 5.1.23) for metabolite matching and qualitative identification with public and local databases (such as MassBank, MetaboBASE, and GNPS). The mass accuracy for MS tolerance was setting at 0.01 Da and 0.025 Da for MS/MS tolerance. The minimum peak height was setting to 8000 amplitude. Peak extraction MS tolerance had a mass tolerance of 15 ppm, and a retention time tolerance of 0.1 min. The identified metabolites should have a detection rate of 60 % or higher in the overall sample. Metabolites with a MS/MS spectrum and MS/MS similarity higher than 0.95 (>0.95) were selected for further analysis.

### 2.7 Statistics analysis

Statistical analysis was performed by SPSS software Version 26.0 (IBM Crop.). Independent-sample *t*-test or one-way ANOVA was used to identify significant differences (*P* < 0.05) upon treatments. Duncan test as post hoc analysis was used for multiple comparisons among group means. The PCA, PLS-DA model, and Pearson rank correlation were performed by R (R Core Team, 2016) with R package mixOmics, ropls, corrplot, and ggplot2 for visualization. *MataboAnalyst* 6.0 (https://www.metaboanalyst.ca) and Cytoscape (version 3.8.2) was performed for the KEGG pathway and network analysis of different expressed metabolites in potatoes.

## 3 Results and discussion

### 3.1 The appearances and basic nutrients in different varieties of potatoes

**Fig. 1:**
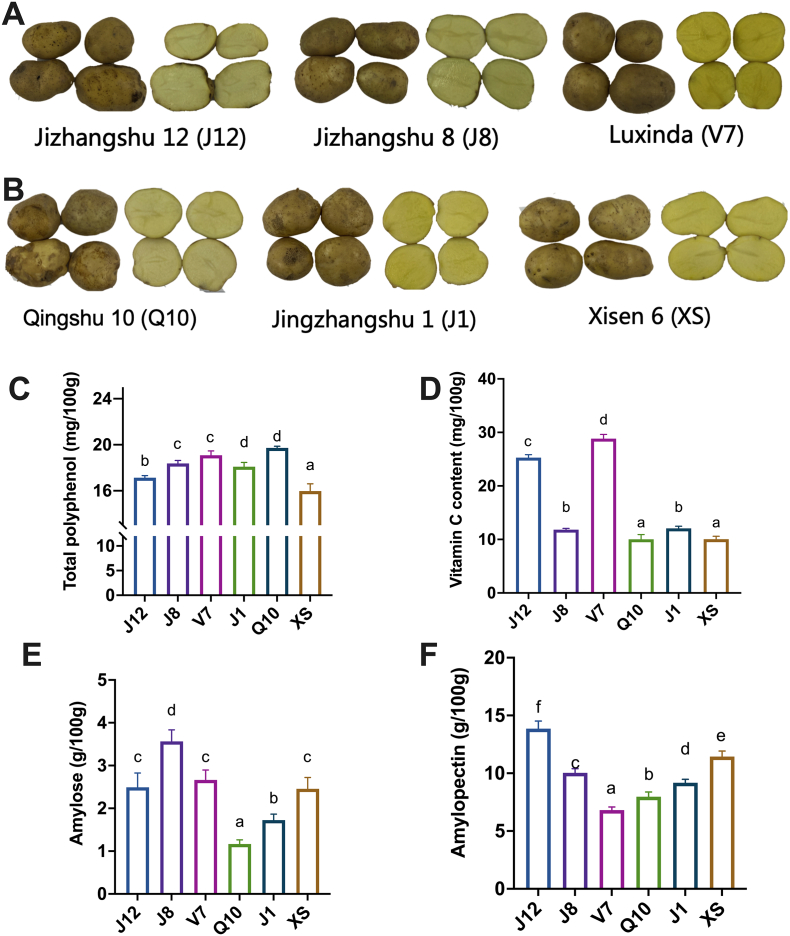
The appearances of six varieties of potato, which are classified as (A) FE, fresh eating; (B) FP, food processing; (C—F) the basic nutrients in different varieties of potatoes.

The appearances and the basic nutrients of six potato varieties are shown in Fig. 1. The six varieties investigated in this study included Jizhangshu 12 (J12), Jingzhangshu 1 (J1), Luxinda (V7), Qingshu 10 (Q10), Jizhangshu 8 (J8), and Xisen 6 (XS), and they were classified as fresh eating (FE) and food processing (FP) types of potato based on the internet database. Generally, the results showed no significantly differences in the appearances of six potato varieties (Fig. 1A, B). The basic nutrients in six varieties of potato are shown in Fig. 1C-1F. As shown in Fig. 1C, the total polyphenol contents in potato varied significantly (*P* < 0.05). For example, the total polyphenol was the highest in Q10 (19.73 ± 0.14 mg/100 g) and the lowest in XS (15.97 ± 0.64 mg/100 g), and these two potatoes belong to FP type. This may reveal that the polyphenol content may not directly related to the processing properties for potato. Moreover, the vitamin C was the most abundant in FE potatoes, particularly in V7 (28.83 ± 0.79 mg/100 g) and J12 (25.29 ± 0.55 mg/100 g) compared to FP potato. As for the starch composition, the results showed that the amylose and amylopectin contents were significantly different among the different varieties of potatoes. The results showed that the amylose content was highest (3.57 ± 0.27 g/100 g) in J8 and being the lowest in Q10 (1.17 ± 0.10 g/100 g). As for amylopectin, J12 contained the highest amylopectin content (13.86 ± 0.66 g/100 g) followed by XS (11.44 ± 0.48 g/100 g) and J8 (10.03 ± 0.37 g/100 g). These results align with previous studies which reported the ratio of amylopectin and amylose was about 3:1 (Jansky & Fajardo, 2016). Moreover, the ratio between amylopectin and amylose were observed being higher in FP potato (5.60,1) compared to FE potato (3.65,1). Previous study reported that higher amylopectin/amylose ratio was related to the pasting, gelatinization, retrogradation, swelling, solubility, digestibility and other processing properties (Tong et al., 2023). Overall, potatoes with different processing properties had large differences in their basic nutrients, and these variations may be influenced by the multiple factors.

### 3.2 Identification of volatile compounds in different varieties of potatoes

**Fig. 2:**
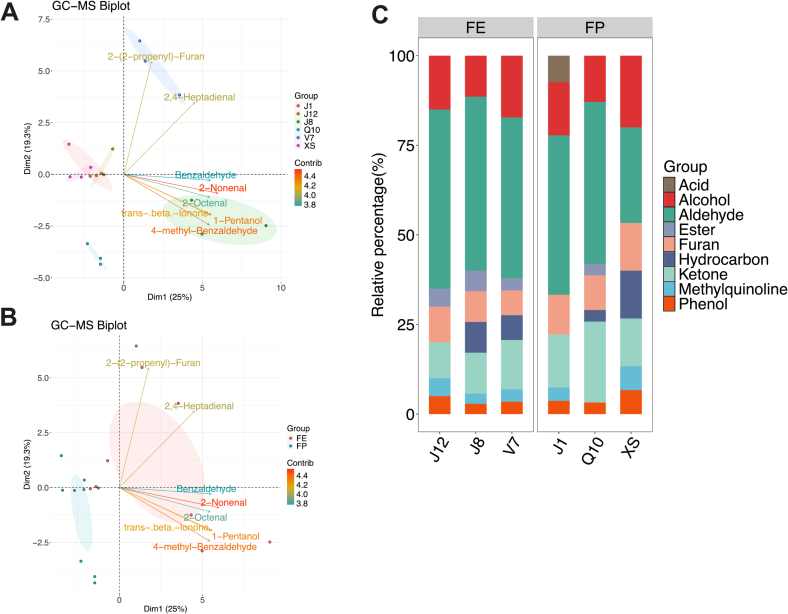
(A) the biplot of volatile compounds in six varieties of potato; (B) the biplot of volatile compounds biplot of volatile compounds in FE and FP varieties of potato; (C) the stacked bar chart of volatile compounds in six varieties of potato. (*n* = 3 per potato variety).

To characterize the differences in volatile compounds among different potato varieties, GC–MS analysis was performed, as shown in Table 1. The results showed that the concentration of 2-Octenal was the most abundant volatile compound in potato followed by 2-pentyl-Furan, 1-Octen-3-ol, and (E,Z)-2,4-Decadienal,. This is reasonable because the flavor of 2-Octenal is descripted as roasted potato. Jiang et al. (2023) demonstrated the abundant of 2-Octenal in several varieties of potatoes in China. A previous study reported that fresh potatoes contained large amounts of 2,4-Decadienal, which is generated by the reaction of unsaturated fatty acids triggered by lipoxygenase after cell rupture (Y. Wu et al., 2023). Besides that, biplots and stacked bar charts were performed to analysis the characteristic compounds and the variations in classifications of volatile compounds among different potato varieties (Fig. 2). Fig. 2A shows PC1 (24.9 %) and PC2 (20.0 %) distinguished the volatile compounds in six potato varieties. Of these, PC1 distinguished the volatile compounds in V7 and J8 with others, and PC2 distinguished volatile compounds in V7 with J8 and Q10. In contrast, J1, J12, and XS were not well distinguished by PC1 and PC2. The loading plot in Fig. 2A shows that the characteristic volatile compounds in V7 potato included 2,4-Heptadienal, 3,5-Octadien-2-one, and 2-(2-propenyl)-Furan. Moreover, 2-Nonenal, 4-methyl-Benzaldehyde, 1-Pentanol, and trans- beta-Ionone were abundant in J8 potato. Furthermore, the volatile compounds in FE and FP potato were distinguished by PC1 (25.0 %) and PC2 (19.3 %). The loading plot in Fig. 2B shows that the volatile compounds were more abundant in FE potato, such as 2-Nonenal, 4-methyl-Benzaldehyde, 1-Pentanol, and trans- beta-Ionone. This is consistent with the fact that FE potato contained more volatile compounds, making them more suitable for direct consumption. For example, 2-Nonenal is a monounsaturated fatty aldehyde which contributes to the flesh aroma for potatoes (Dresow & Böhm, 2009).

**Table 1:** The identification of volatile compounds in 6 varieties of potato (n = 3).

Volatile compound (μg/g)	Chemical formula	Molecular weight	Description	J8	J12	J1	Q10	V7	XS
methyl-Cyclopentane	C6H12	84.094	Petrol-like	0.04 ± 0.01	–	–	–	0.05 ± 0.00	0.12 ± 0.09
n-Hexane	C6H14	86.11	Petrol-like	0.30 ± 0.08^a^	–	–	–	2.56 ± 1.84^b^	0.31 ± 0.03^a^
Hexanal	C6H12O	100.089	Pungent smell	0.01 ± 0.00	0.03 ± 0.04	0.05 ± 0.08	0.00 ± 0.00	0.25 ± 0.25	0.02 ± 0.02
2-Pentenal	C5H8O	84.058	Rubber smell	0.05 ± 0.04	0.02 ± 0.02	0.02 ± 0.00	–	–	–
2-n-Butyl furan	C8H12O	124.089	Smell as sweet	0.05 ± 0.03	0.03 ± 0.01	0.17 ± 0.22	0.07 ± 0.02	–	0.02 ± 0.02
2-pentyl-Furan	C9H14O	138.104	Off-flavor, sweetness, boiled smell	2.24 ± 0.49^b^	0.69 ± 1.15^a^	0.73 ± 0.26^a^	2.55 ± 1.12^b^	1.43 ± 0.77^ab^	0.37 ± 0.32^a^
2-Octenal	C8H14O	126.104	Roasted potato flavor, boiled smell, fatty	6.85 ± 1.75^d^	0.87 ± 0.00^ab^	1.28 ± 0.42^abc^	2.48 ± 1.39^bc^	3.19 ± 1.52^c^	0.19 ± 0.13^a^
1-Octen-3-ol	C8H16O	128.12	Lightly smell, herb smell, lavender smell	0.85 ± 0.04^b^	0.90 ± 0.00^bc^	0.42 ± 0.09^a^	0.47 ± 0.09^a^	1.09 ± 0.07^c^	0.49 ± 0.26^a^
2,4-Heptadienal	C7H10O	110.073	Rancid smell	0.85 ± 0.05^e^	0.50 ± 0.00^c^	0.64 ± 0.06^d^	0.19 ± 0.07^b^	1.01 ± 0.03^f^	–
Benzaldehyde	C7H6O	106.042	Bitter almond smell	1.09 ± 0.20^b^	–	–	0.20 ± 0.03^a^	0.67 ± 0.78^ab^	–
2,4-Octadienal	C8H12O	124.089	Pear-like smell	0.34 ± 0.21^b^	–	0.13 ± 0.04^a^	–	0.20 ± 0.03^ab^	–
(E, E)-3,5-Octadien-2-one	C8H12O	124.089	Fruity, grassy-like smell	0.12 ± 0.08^b^	–	0.04 ± 0.00^a^	0.04 ± 0.00^a^	–	–
2-methyl-Benzaldehyde	C8H8O	120.058	Cherry-like smell	0.05 ± 0.00	–	–	0.05 ± 0.01	–	–
Benzeneacetaldehyde	C8H8O	120.058	Sweet fruity, hyacinth- like smell	0.17 ± 0.07^a^	0.34 ± 0.00^b^	0.19 ± 0.07^a^	1.01 ± 0.02^c^	0.33 ± 0.04^b^	0.19 ± 0.10^a^
Acetophenone	C8H8O	120.058	Fruity, hawthorn-like smell	0.02 ± 0.01^a^	–	–	0.03 ± 0.01^a^	0.17 ± 0.12^b^	0.04 ± 0.01^a^
(E, E)-2,4-Nonadienal	C9H14O	138.104	Chicken soup, fatty, flower, fruit-like smell	0.19 ± 0.16	0.10 ± 0.11	0.17 ± 0.11	0.03 ± 0.00	0.14 ± 0.07	–
1,2-dimethoxy-Benzene	C8H10O2	138.068	Sweet and creamy smell	0.02 ± 0.01	–	–	0.01 ± 0.00	0.02 ± 0.01	–
(E, Z)-2,4-Decadienal2	C10H16O	152.12	Wax, geranium-like smell	0.49 ± 0.42	0.58 ± 0.43	2.52 ± 2.61	0.24 ± 0.12	0.13 ± 0.02	–
Methyl salicylate	C8H8O3	152.047	Wintergreen smell	0.24 ± 0.06	0.48 ± 0.59	–	–	0.34 ± 0.02	–
(E, E)-2,4-Decadienal	C10H16O	152.12	Wax, geranium-like smell	0.34 ± 0.10	0.42 ± 0.00	2.31 ± 2.64	0.19 ± 0.09	0.02 ± 0.04	–
Mequinol	C7H8O2	124.052	Caramel smell	0.30 ± 0.09^bc^	0.14 ± 0.09^ab^	0.07 ± 0.05^ab^	–	0.18 ± 0.11^abc^	0.04 ± 0.01^a^
Benzyl alcohol	C7H8O	108.058	Lightly aromatic smell	0.28 ± 0.05^ab^	0.30 ± 0.00^b^	0.36 ± 0.02^bc^	0.63 ± 0.02^d^	0.16 ± 0.05^a^	0.45 ± 0.15^c^
Phenylethyl Alcohol	C8H10O	122.073	Lightly rose-like smell	0.25 ± 0.07^b^	–	0.06 ± 0.01^a^	0.28 ± 0.06^b^	0.03 ± 0.02^a^	0.02 ± 0.01^a^
4-(2,6,6-trimethyl-1-cyclohexen-1-yl)-3-Buten-2-one	C13H20O	192.151	Violet smell	0.01 ± 0.00	0.02 ± 0.01	–	0.01 ± 0.00	0.02 ± 0.01	–
1-Pentanol	C5H12O	88.089	Lightly smell	0.12 ± 0.03^b^	0.05 ± 0.02^a^	0.05 ± 0.00^a^	0.05 ± 0.00^a^	0.05 ± 0.02^a^	–
cis-2-(2-Pentenyl) furan	C9H12O	136.089	–	0.11 ± 0.03^a^	–	–	0.20 ± 0.05^b^	–	–
2-Heptenal	C7H12O	112.089	Almond, fatty smell	0.18 ± 0.04	–	–	–	–	–
Nonanal	C9H18O	142.136	Strong fatty smell, rose and citrus-like smell	0.05 ± 0.02	0.03 ± 0.00	–	0.03 ± 0.00	–	–
Copaene	C15H24	204.188	Honey smell	0.04 ± 0.00	–	–	0.03 ± 0.01	–	–
2-Nonenal	C9H16O	140.12	Fatty, aldehyde, citrus-like smell	0.30 ± 0.07^b^	–	0.13 ± 0.01^a^	0.13 ± 0.00^a^	0.19 ± 0.10^a^	–
3,5-Octadien-2-one	C8H12O	124.089	Fruity, grassy-like smell	0.16 ± 0.04^a^	–	0.04 ± 0.01^a^	0.04 ± 0.01^a^	–	–
4-methyl-Benzaldehyde	C8H8O	120.058	Almond and flower-like smell	0.15 ± 0.04^b^	–	–	0.03 ± 0.02^a^	–	0.03 ± 0.01^a^
trans-.beta.-Ionone	C13H20O	192.151	Violet smell	0.10 ± 0.03^b^	0.02 ± 0.01^a^	0.03 ± 0.01^a^	0.02 ± 0.00^a^	0.03 ± 0.00^a^	–
Dimethyl phthalate	C10H10O4	194.058	Lightly aromatic smell	0.02 ± 0.01^b^	–	–	0.01 ± 0.00^a^	–	–
2-methoxy-Phenol	C7H8O2	124.052	Lightly phenol smell	0.21 ± 0.18	0.07 ± 0.10	0.11 ± 0.06	0.37 ± 0.12	0.12 ± 0.16	0.04 ± 0.01
1-Penten-3-one	C5H8O	84.058	Irritating smell	–	–	0.15 ± 0.00^a^	0.55 ± 0.17^b^	–	–
2-(2-propenyl)-Furan	C10H12ClN5O3	108.058	–	–	–	0.06 ± 0.00^a^	–	0.17 ± 0.01^b^	–
2-Hexenal	C6H10O	98.073	Fresh leaf, fruity-like sell	–	–	0.02 ± 0.01	–	0.02 ± 0.01	–
3-Octen-2-one	C8H14O	126.104	Spicy smell	–	–	–	0.04 ± 0.00^a^	0.09 ± 0.03^b^	0.06 ± 0.03^ab^
Octanoic acid	C8H16O2	144.115	Bad smell	–	–	0.25 ± 0.12	–	–	–
2-Octenoic acid	C8H14O2	142.099	–	–	–	0.17 ± 0.02	–	–	–
2,4-Nonadienal	C9H14O	138.104	Chicken soup, fatty, flower, fruit-like smell	0.04 ± 0.00	0.03 ± 0.01	0.09 ± 0.10	0.04 ± 0.00	0.09 ± 0.09	–
3-Nonen-1-ol	C9H18O	142.136	–	–	–	–	–	0.27 ± 0.05	–

All values are shown as means ± SD. The superscript letters indicate results of One-way ANOVA and Duncan test as post hoc for multiple comparisons with significant level at *P* < 0.05. Different lowercase letters indicate significant differences of volatile compounds (μg/g) between different varieties of potatoes.

The stacked bar chart was used to identify the composition of volatile compounds in different potato varieties (Fig. 2C). Of these, aldehyde compounds were the most abundant category in potato, which is in line with previous study about the volatile compound composition in potato (Dresow & Böhm, 2009). FE potato contained a higher proportion of aldehyde and alcohol compounds compared to FP potato, while FP potatoes were observed to be abundant in hydrocarbon, furan, and ketone compounds. The higher amounts of aldehyde and alcohol compounds in FE potatoes reveal more strong potato characteristics aroma, which may be more appealing to customers (Dresow & Böhm, 2009). Overall, GC–MS results showed significant differences in volatile compounds among potatoes with different processing properties. Several aldehyde and alcohol compounds such as 2-Nonenal, 4-methyl-Benzaldehyde, and 1-Pentanol were considered as characteristic compounds to contribute the unique aroma property for FE potatoes, and thus could be used to identify the FE potato.

### 3.3 Metabolite composition in different varieties of potatoes

**Fig. 3:**
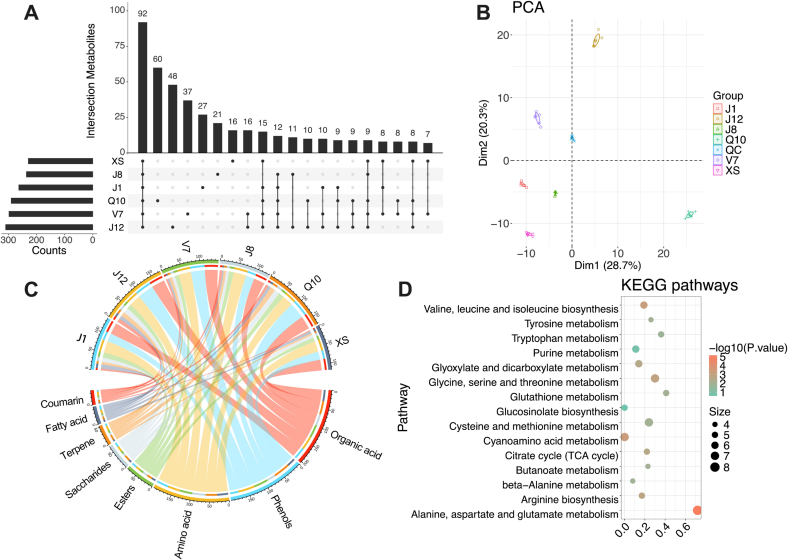
(A) the Upset R plot of metabolites in six varieties of potato; (B) PCA analysis of metabolites in six varieties of potato; (C) the dominant classification of metabolites in six varieties of potato; (D) KEGG analysis of metabolites in six varieties of potato. (*n* = 6 per potato variety).

Metabolomics analysis was performed to compare the different metabolite compositions between different potato varieties. Fig. 3 presents an overview of metabolites in different varieties of potatoes. The results showed that six potato varieties had 92 metabolites in common, and J12 contained the highest amounts (312) of metabolites but XS contained the lowest number (231) of metabolites (Fig. 3A). Moreover, Q10 contained the highest amounts (60) of unique metabolites followed by J12 (48) and V7 (37). Fig. 3B shows that PCA analysis distinguished the six potato varieties, with QC samples clustered into one group located in the middle of PCA. It reveals that the reliability of metabolomics data (Broeckling et al., 2023). The dominant metabolite categories in potato included organic acid, phenols, amino acid, which is line with previous study (Chaparro et al., 2018). The chord diagram in Fig. 3C shows the large differences in metabolite compositions among different potato varieties. For example, J12 and Q10 contained the highest amounts (45) of phenols, and J1 (23), XS (23), J8 (24) contained the lower numbers of that. Higher contents and amounts of phenol in potato presented antioxidant property (Kim et al., 2019). Moreover, the numbers of organic acids and amino acids were similar across different potato varieties. To understand the biological functions of these metabolites in potato, KEGG analysis was performed, as shown in Fig. 3D. The results showed that most of metabolites were involved in alanine, aspartate and glutamate metabolism, cyanoamino acid metabolism, and glycine, serine and threonine metabolism. The alanine, aspartate and glutamate metabolism in plants has been reported as the intermediates of central metabolism such as the citric acid cycle (Kim et al., 2019). Plant amino acid metabolism provides signaling molecules, defense compounds, and nutrients to shape interactions with microbes (Moormann et al., 2022). Moreover, cyanoamino acid metabolism in plant is involved in the biosynthesis of cyanogenic glycosides, which act as a defense mechanism against herbivores and pathogens. This reveals that most of metabolites in the tuber organogenesis of potato are derived from interactions with external environment especially soil. Overall, the large differences in metabolites were observed among different varieties of potatoes, and these variations may result in the changes in their functions and processing properties.

### 3.4 Correlation analysis of volatile compound and metabolites in potato

**Fig. 4:**
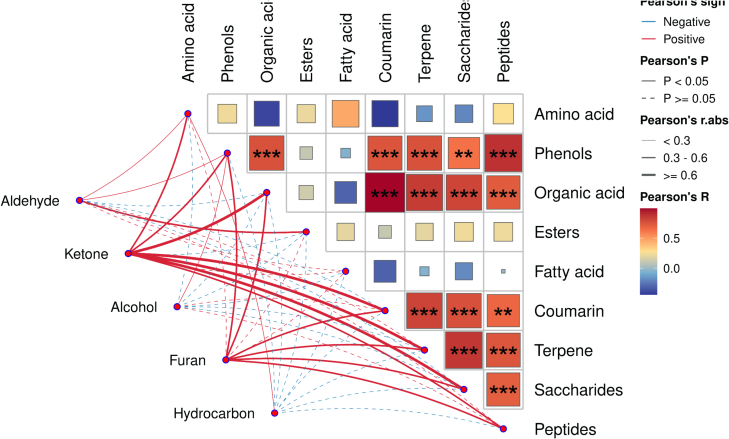
The Pearson analysis of dominant classifications of volatile compound with the dominant classifications of metabolite.

To reveal the potential relationships between volatile compounds and metabolites, Pearson correlation analysis between dominant volatile compound and metabolites was performed, as shown in Fig. 4. The results showed that the contents of ketone compounds were positively correlated with the contents of organic acids (R^2^ = 0.81), coumarin (R^2^ = 0.79), terpene (R^2^ = 0.74) and being correlated with saccharides (R^2^ = 0.65), phenols (R^2^ = 0.55), and amino acids (R^2^ = 0.40). Previous study have reported the biosynthesis of ketones was related to the coumarin derivatives, and the higher amounts of coumarin compounds may results in the larger amounts of accumulations of ketone compounds (Loncaric et al., 2020). Furthermore, the correlation between ketone and terpene is easily explained, as terpene compounds can be divided into alcohols, aldehydes, esters, ether, epoxides, and ketones. Moreover, the results showed that the contents of aldehyde compound were positively correlated with esters (R^2^ = 0.55) followed by phenols (R^2^ = 0.24), and amino acid (R^2^ = 0.22). This is also reasonable because both aldehyde volatile compounds and ester metabolites were catalyzed and synthesized by the acyl-CoA (Rey-Serra et al., 2022), and aldehyde compound can be oxidized to form esters (Kunjapur & Prather, 2015). Aldehydes have been found to be related to the degradation processes of amino acids, fatty acids, and other substances in sweet potato (Yao et al., 2024). As for the contents of furan compounds, the results indicated the positive correlations with saccharides (R^2^ = 0.57), organic acid (R^2^ = 0.50), coumarin (R^2^ = 0.46), terpene (R^2^ = 0.40), and phenols (R^2^ = 0.34). Istasse and Richel (2021) demonstrated that the synthesis of furan and its derivatives were through acid-catalyzed dehydration of saccharides. This reveals that large amounts of saccharides served as substrates for the synthesis of furan derivatives in potatoes. The results showed that aforementioned volatile compounds were contributed by the combinations of several metabolite categories, while contents of hydrocarbon compound were slightly correlated with amino acid (R^2^ = 0.16).

Besides that, the results also showed correlations between metabolite categories. For example, strong correlations were observed between phenols with peptides (R^2^ = 0.89), terpene (R^2^ = 0.76), organic acid (R^2^ = 0.76), coumarin (R^2^ = 0.75), saccharides (R^2^ = 0.63). Previous study reported that the interactions between phenols with peptides through non-covalent bonds and formed the polyphenol-protein/peptide complexes (Pérez-Gregorio et al., 2020). Similar correlations between organic acid with coumarin (R^2^ = 0.99), terpene (R^2^ = 0.85), saccharides (R^2^ = 0.81), peptides (R^2^ = 0.72) were found. Moreover, strong correlations were also observed between coumarin with terpene (R^2^ = 0.82), saccharide (R^2^ = 0.77), and peptide (R^2^ = 0.67); terpene with saccharide (R^2^ = 0.86) and peptide (R^2^ = 0.74); saccharide with peptide (R^2^ = 0.71). These correlations were mainly due to the plant stress tolerance such as reverse abnormalities back to normal, prevent illnesses, or allay symptoms (Awuchi, 2019). For example, phenols and terpenes have been reported to act against herbivores and pathogens such as gram-negative *Escherichia coli* and gram-positive *Staphylococcus aureus* bacteria (Ergüden, 2021). Environments factors including temperature, soil microorganism, and mineral contents play critical roles in shaping the nutrient components of plants (Gutiérrez-Quequezana et al., 2020; Hemkemeyer et al., 2024; Starovoitova et al., 2021). However, in this study, the potatoes were collected from the same area, ensuring consistent environmental conditions. Therefore, the large variations of metabolites in different varieties of potato may be caused by the different genomes (Gutiérrez-Quequezana et al., 2020), thus resulted in the changes in their volatile compounds and processing properties. Overall, the interaction networks between metabolites and volatile compound in potatoes were observed, and these results will provide more dataset for selective breeding for potato in the future.

### 3.5 Different expression of metabolites among different varieties of potatoes

**Fig. 5:**
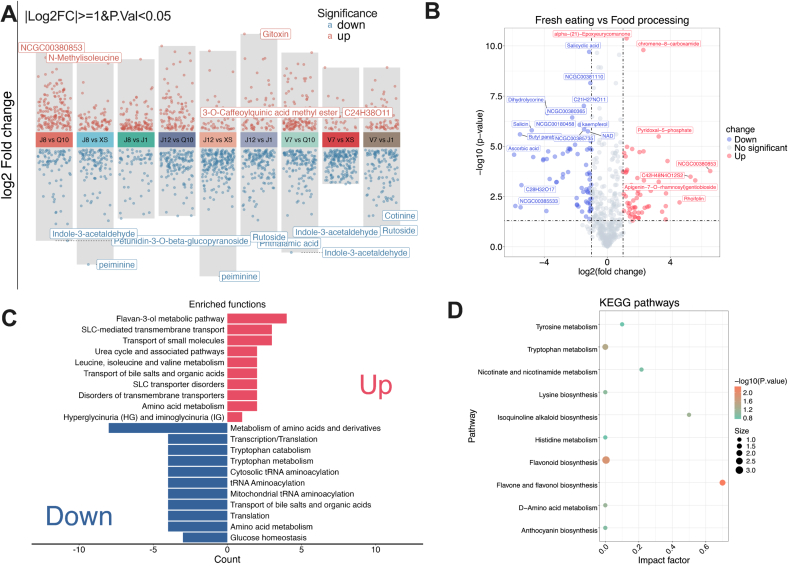
(A) the volcano plot of different expressed metabolites between individual FE and FP potato; (B) the volcano plot of different expressed between all FE and FP potato; (C) the enriched functions of different expressed between FE and FP potato; (D) the KEGG analysis of different expressed metabolites between FE and FP potato. (n = 6 per potato variety).

The different expressions and KEGG analysis of metabolites between potatoes with different processing property, as shown in Fig. 5. Fig. 5A shows the individual comparisons between FE potato with FP potato. The results indicated significant differences between potatoes with different processing properties. To better compare the different expressions of metabolite among potato with different processing property, the volcano plot was performed between FE potato and FP potato, as shown in Fig. 5B. In line with the results from Fig. 5A, large differences in metabolites were observed. Generally, compared to FE potatoes, higher amounts of metabolites were down-regulated in FP potatoes. For example, compared to FE potato, FP potato contained higher intensity of apigenin-7-O-rhamnosyl gentiobioside, rhoifolin, trimethyl-6-methylidene-10-oxatetracyclo hexadecane, 3-hydroxybutanoic acid, and ilicic acid. Rhoifolin is a common and important flavonoid in the plant, which has been reported to have antioxidant, anti-inflammatory, antimicrobial, hepatoprotective, and anticancer properties (Refaat et al., 2015). Moreover, 3-hydroxybutanoic acid has been reported being involved in the synthesis and degradation of ketone bodies in the plant (Mierziak et al., 2021). In contrast, salicin, petunidin-3-O-beta-glucopyranoside, butyl paraben, ascorbic acid and other metabolites were down-regulated in FP potato. Of these, slaicin releases as salicylic acid, which is considered as an endogenous signal mediating local and response for plant defense against pathogens (Rivas-San Vicente & Plasencia, 2011). Ascorbic acid is considered as a major redox buffer and as a cofactor for enzymes in plant, which has been reported being involved in regulating photosynthesis, hormone biosynthesis, and regenerating other antioxidants (Gallie, 2013).

To understand the variations in biological functions of these differently expressed metabolites, enriched functions and KEGG analysis was performed in Fig. 5C and D. Fig. 5C indicated that up regulated metabolites in FP potato were mainly associated with flavan-3-ol metabolic pathway, SLC-mediated transmembrane transport, and transport of small molecules. Furthermore, the down-regulated metabolites in FP potato were mainly related to biochemical pathways, metabolism of amino acids and derivatives, transcription/translation. Tryptophan catabolism and other functions. The KEGG analysis showed that these different expressed metabolites were mainly involved in flavone and flavanol biosynthesis, flavonoid biosynthesis, tryptophan metabolism, and isoquinoline alkaloid biosynthesis. The results showed that most of differently expressed metabolites were involved in flavonoid related biosynthesis pathway. Previous study reported that flavonoids were generated by numerous pathways such as the pentose phosphate pathway, glycolysis pathway, shikimic acid pathway, and phenylpropane pathway (X. Wu & Xiao, 2024). Flavonoids were mainly involved in stress responses, signaling, protection against ultraviolet radiation and phytopathogens, male fertility, and auxin transport (Casati, 2012). Moreover, tryptophan metabolism plays a critical role in the regulation of plant growth and development, stress responses, and synthesis of numerous bioactive molecules such as auxin, tryptamine derivatives, and terpenoid indole alkaloids (Yang et al., 2020). Previous studies reported that several amino acids such as cysteine, glutamic acid, and valine could consistently reduce the browning process for potato after fresh-cut (Ali et al., 2016; Song et al., 2023). This aligns with the results in this study, which highlight the critical roles of amino acid metabolism in determine the processing properties of potato. Overall, the different expressed of metabolites between FP and FE potatoes were studied, and these metabolites may play critical roles in the composition of potatoes with different processing properties.

### 3.6 Comparison analysis of metabolite and volatile compound in potato with different processing properties

**Fig. 6:**
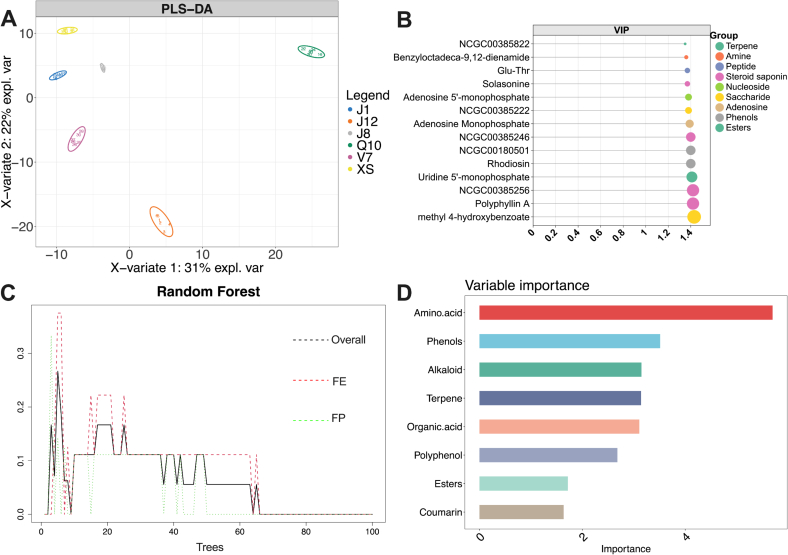
(A) the PLS-DA model of metabolites in six varieties of potato; (B) the metabolite with top 14 VIP value from PLS-DA; (C) the OBB error of random forest; (D) the top 8 important variables from random forest.

The PLA-DA model was used to identify the characteristic metabolites among different varieties of potatoes, combining variables from basic nutrients, volatile compounds, and metabolites (Fig. 6A and B). The R^2^ and Q^2^ values of the PLS-DA model were used to evaluate the fitting degree of the model (Fig. S1A). The results showed the greatly fitness and predictability of PLS-DA model for six varieties of potato, with R^2^X = 0.885, R^2^Y = 0.997, and Q^2^ = 0.978. Fig. 6A shows that the volatile compounds and metabolites are significantly different among six varieties of potato. Moreover, the VIP values for dominant compounds to distinguish the varieties of potato are calculated in the PLS-DA model. The top 14 metabolites with highest VIP value are shown in Fig. 6B, and the main categories includes steroid saponin and phenols.

The random forest model was used to identify the important variables distinguishing the potato processing properties especially FE and FP. The variables included the basic nutrients, volatile compounds, and metabolites. The classification trees for these top 10 metabolite categories were set to 100. The cumulative classification error (OOB error) steadily dropped as the number of trees rose and finally stabilized at 0.000 (Fig. 6C). The top 8 important variables in random forest classification model were shown in Fig. 6D, and these variables were impacted considerably to the accuracy of the random forest classification. As similar as previous results, amino acid plays critical role in identification of the processing properties for potato. This is in line with previous study about genome-wide association studies about the critical role of amino acid distribution between FE potato with FP potato (Pandey et al., 2023).

**Fig. 7:**
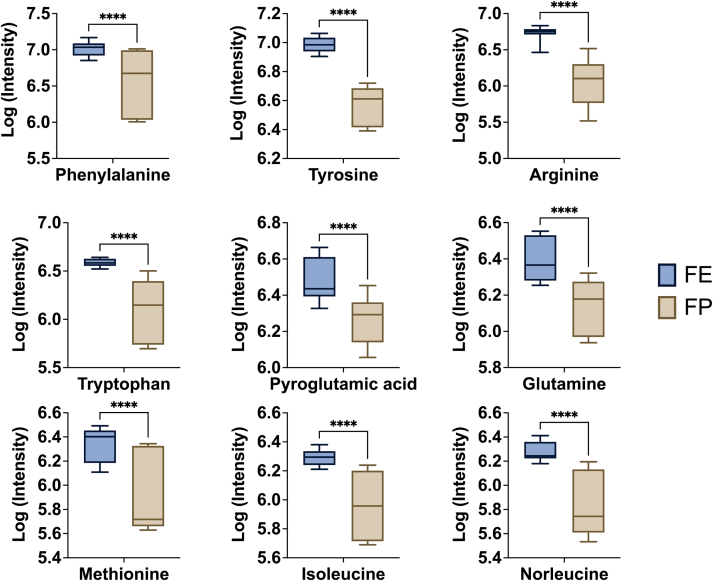
The comparison analysis of dominant amino acids in FE and FP potato. (n = 6).

The box plots of dominant metabolites as amino acids are shown in Fig. 7. It is clearly that these amino acids were higher in FE potato compared to FP potato (*P* < 0.05), confirming the aforementioned results. Among these, phenylalanine, tyrosine, and tryptophan as the most dominant amino acids belong to aromatic amino acids. These aromatic amino acids in FE potatoes are important to human health because these amino acids serve as precursors for the synthesis of many biologically/neurologically active compounds (Han et al., 2019). Several charged amino acids such as arginine, lysine, and aspartic acid affected the physical and chemical characteristics of starch-based products derived from potatoes such as swelling power, solubility, light transmittance, and gel strength of the potato starch (Cui et al., 2014). Glutamine, glutamic and aspartic acid, serine, valine, and proline play critical roles in determining the taste of potato tubers (Pandey et al., 2023). Moreover, the methionine content in potato is interested to related industry because of the related nutritional and fragrance benefits (Pandey et al., 2023). The differences in amino acid related metabolisms were caused by the large differences in their genomes. Previous study about the metabolite and genome analysis of potato species also reported that slight differences in amino acid composition caused considerable variation in the catalytic properties of the enzyme, which may mainly determine the metabolite composition and volatile compounds in potatoes (Aversano et al., 2017). Overall, several metabolites including amino acid, phenols, and alkaloid were characterized as the important variables to identify the processing varieties of potatoes. These amino acids are involved in many metabolism pathways such as enzyme synthesis, thus alter the aroma and taste of potatoes with different processing properties.

## 4 Conclusion

In conclusion, this study identified the basic nutrients, volatile compounds, and metabolite composition in six varieties of potatoes. The results showed significant differences in basic nutrients, volatile compounds, and metabolite composition among different varieties of potatoes, and some correlations between volatile compounds and metabolites were also observed. Moreover, the differences in volatile and metabolite compounds were compared between FE and FP potatoes. Compared to FP potato, FE potato contained higher proportion of aldehyde and alcohol compounds, but being lower in hydrocarbon, furan, and ketone compounds. The volcano plot, enriched functions, and KEGG analysis showed that the different expressed metabolites were involved in biochemical pathways, metabolism of amino acid and its derivatives, flavone and flavanol biosynthesis, and tryptophan metabolism. Furthermore, the random forest model showed that the FE and FP potatoes could be distinguished by the contents of amino acid and phenols. Together, these results will contribute a novel understanding of the relations between volatile compounds and metabolites in potato with different processing properties and help the selective breeding of potato in food industries.

## CRediT authorship contribution statement

**Wenyuan Zhang:** Writing – original draft, Software, Investigation, Formal analysis, Conceptualization. **Liang Li:** Methodology, Formal analysis. **Yaqi Zhao:** Formal analysis. **Haixia Yang:** Writing – review & editing. **Xuejie Zhang:** Writing – review & editing. **Zhanquan Zhang:** Writing – review & editing. **Xue Wang:** Methodology. **Zhenzhen Xu:** Methodology. **Wanxing Wang:** Writing – review & editing, Resources, Methodology, Investigation, Funding acquisition, Conceptualization. **Jianjun Deng:** Writing – review & editing, Project administration, Investigation, Funding acquisition, Conceptualization.

## Declaration of competing interest

The authors declare that they have no known competing financial interests or personal relationships that could have appeared to influence the work reported in this paper.

## Data Availability

Data will be made available on request.

## Appendix A Supplementary data

Figure S1 (A) The fitting degree of PLS-DA model; the heatmap about the intensity of top 14 metabolites Image 1
