# Supplementary material for: Characterization of differences in volatile compounds and metabolites of six varieties of potato with different processing properties
Source: Food Chem X. 2024 Dec 22;25:102116. doi: 10.1016/j.fochx.2024.102116 (PMC11741029; doi:10.1016/j.fochx.2024.102116)

Figure S1 (A) The fitting degree of the PLS-DA model; the heatmap about the intensity of top 14 metabolites.


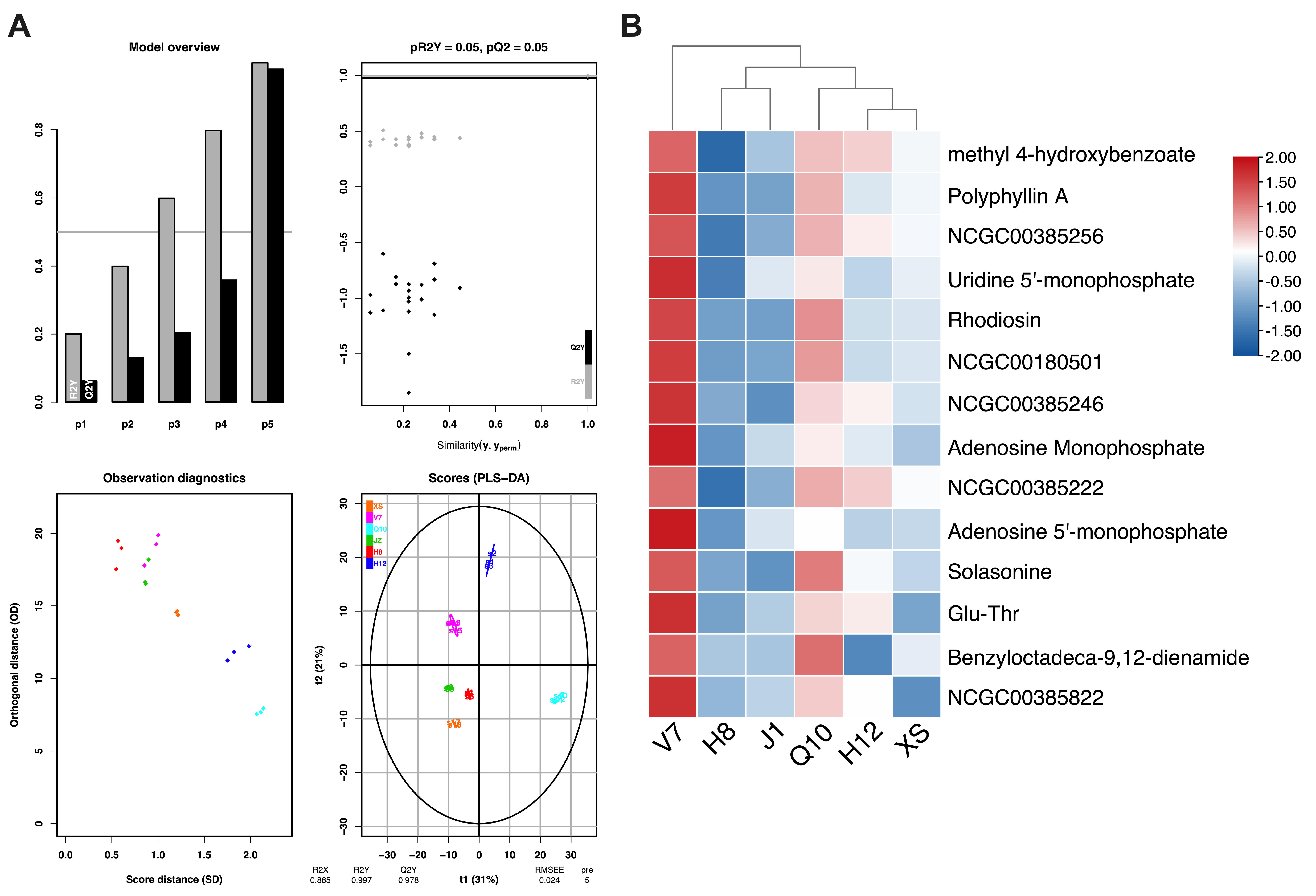

Supplement: Supplementary file 1 — Figure S1 (A) The fitting degree of PLS-DA model; the heatmap about the intensity of top 14 metabolites [file mmc1.docx]
